# The Analysis of Chitosan-Coated Nanovesicles Containing Erythromycin—Characterization and Biocompatibility in Mice

**DOI:** 10.3390/antibiotics10121471

**Published:** 2021-11-30

**Authors:** Loredana Nicoleta Hilițanu, Liliana Mititelu-Tarțău, Grațiela Eliza Popa, Beatrice Rozalina Buca, Liliana Lăcrămioara Pavel, Ana-Maria Pelin, Andreea-Daniela Meca, Maria Bogdan, Daniela Angelica Pricop

**Affiliations:** 1 Department of Pharmacology, Faculty of Medicine, “Grigore T. Popa” University of Medicine and Pharmacy, 700115 Iasi, Romania; ln.rusu@yahoo.com (L.N.H.); beatrice-rozalina.buca@umfiasi.ro (B.R.B.); 2 Department of Pharmaceutical Technology, Faculty of Pharmacy, “Grigore T. Popa” University of Medicine and Pharmacy, 700115 Iasi, Romania; eliza.popa@umfiasi.ro; 3 Department of Morphological and Functional Sciences, Faculty of Medicine and Pharmacy, “Dunărea de Jos” University, 800010 Galați, Romania; doctorpavel2012@yahoo.com; 4 Department of Pharmaceutical Sciences, Faculty of Medicine and Pharmacy, “Dunărea de Jos” University, 800010 Galați, Romania; anapelin@gmail.com; 5 Department of Pharmacology, Faculty of Pharmacy, University of Medicine and Pharmacy, 200349 Craiova, Romania; andreea_mdc@yahoo.com; 6 Department of Physics, Faculty of Physics, “Al. I. Cuza” University, 700506 Iasi, Romania; daniela.a.pricop@gmail.com

**Keywords:** nanovesicles, erythromycin, chitosan, biocompatibility, mice

## Abstract

Nanoantibiotics have proved improved pharmacokinetic characteristics and antimicrobial features. Recent studies have shown non-toxicity, non-immunogenicity, antioxidant, anti-hyperlipidemic, and hepatocyte protective actions, among other advantages of chitosan-based nanoparticles. The purpose of our study was the structural analysis of novel chitosan-coated vesicles entrapping erythromycin (ERT) and the assessment of their biocompatibility in mice. According to the group in which they were randomly assigned, the mice were treated orally with one of the following: distilled water; chitosan; ERT; chitosan vesicles containing ERT. Original nanosystems entrapping ERT in liposomes stabilized with chitosan were designed. Their oral administration did not produce sizeable modifications in the percentages of the leukocyte formula elements, of some blood constants useful for evaluating the hepatic and renal function, respectively, and of some markers of oxidative stress and immune system activity, which suggests a good biocompatibility in mice. The histological examination did not reveal significant alterations of liver and kidney architecture in mice treated with chitosan liposomes entrapping ERT. The results indicate the designed liposomes are a promising approach to overcome disadvantages of conventional ERT treatments and to amplify their benefits and can be further studied as carrier systems.

## 1. Introduction

Antibiotic resistance is one of the biggest threats to global health nowadays and an increasing number of infections (e.g., pneumonia, gonorrhoea, tuberculosis, salmonellosis) are becoming harder to treat as the antibiotics used to cure them become less effective [1].

Novel and effective strategies against multidrug resistant bacteria, are urgently needed. A promising alternative to the search of new therapies are nanoantibiotics, the combination of approved antibiotics with the use of nanotechnology. Recent advances in this domain have enabled the development of drug delivery systems with improved pharmacokinetic characteristics and antimicrobial features [2].

Nanoantibiotics are smaller than 100 nm and it was shown bacteria are less susceptible to develop resistance when exposed to them. Compared to the multiple doses in a systematic release needed for many conventional antibiotics, nanoantibiotics have the advantage of a target-specific, controllable sustained release in a single dose administration [3].

Nanoantibiotics have proved increased drug stability, targeted delivery, prolonged retention, sustained release, and improved penetrating capability. Antibiotic nanoparticles are emerging as a strategy capable of overcoming disadvantages of conventional antimicrobial treatments and amplifying their benefits, by localized delivery to infection sites, diminishing off-target effects, and reducing resistance development [4].

Liposomal nanoformulations can be safely administered by different routes of administration [5], and they can also encapsulate both hydrophobic and hydrophilic drugs, therefore representing one of the most encouraging approaches to deliver antibiotics [6].

Up to date, nanoantibiotic approaches have been made using various well known antibiotics (betalactames, fluoroquinolones, aminoglicosides, macrolides) as metal and metal oxide nanoparticles (silver, gold, zinc oxide, iron oxide), which proved a synergic activity between the antibiotic and the metal involved [7]. Besides metallic nanoparticles, some organic materials have been studied for nanoantibiotic formulation, such as gelatin, chitosan, and peptides, targeting the cell membranes. Compared to metallic nanoparticles, the latter systems have a slow body clearance, are non-toxic and have a long shelf life [8].

One of the modern pharmacotherapy challenges is the use of nanotechnology to optimize drug forms and limit their liver toxicity. The important technological advantages of nanoparticles used as drug delivery systems are represented by targeted release of the active substance, prolongation of the drug’s action in the target tissue, and reduction of their adverse effects [9].

Liposomes are spherical lipid vesicles which contain a central aqueous compartment surrounded by a membrane composed mainly of phospholipids. They are biocompatible, biodegradable, non-immunogenic, and non-toxic [10,11] and have gained increasing attention for their applications as drug delivery systems. In these arrangements they present a series of limitations due to their tendency to aggregate/merge, leading to drug leakage during storage [12]. Moreover, in the case of using such drug carrier systems there is a risk of rapid blood clearance after intravenous injection [13]. To overcome these problems, surface modification processes have been developed by coating the liposomes with various polymers to improve their stability, prolong their life in the blood stream, and provide prolonged release of the entrapped drug [14,15,16].

Various biocompatible and biodegradable polymers, or some natural compounds, have aroused great interest and researchers have investigated their potential as drug carriers with possible biomedical applications.

In the strategy of targeting drugs to increase their absorption, methods have been developed to reduce the absorption of liposomes by cells of the reticuloendothelial system. One of these methods of coating unilamellar lipid vesicles with hydrophilic substances, such as chitosan, has led to reduced phagocytic absorption or minimal interaction with other proteins [17]. Coating these colloidal carriers has been shown to improve particle stability and improve oral transmucosal transport [18]. It has been proven that the most efficient technique for obtaining drug carriers based on lipids is the process of homogenization by sonication, which achieves a narrow distribution of particle size, a higher content of particles in dispersions, avoiding organic solvents. Ensuring good physical stability, dispersibility, and the presence of colloids in the blood circulation are major benefits for systemic use [19].

Chitosan, a polysaccharide of natural origin, obtained from marine crustaceans, mollusks, insects, and fungi, has long attracted attention, especially as a biomedical material [20,21,22], due to its antitumor, antiulcer, immunostimulatory, antidiabetic, antioxidant, and antibacterial activities [15,23]. It can be processed into different forms, such as solutions, gels, blends, sponges, tablets, membrane, paste, for different applications [24].

Chitosan nanoparticles have several advantages: efficiency, cost-effectiveness, biocompatibility, biodegradability, non-toxicity, and non-immunogenicity [24,25]. The use of chitosan-based nanoparticles has proved strong antioxidant, anti-hyperlipidemic and hepatocyte protective actions against the effects of alcohol and fat diet [26]. Literature data have shown favorable results of chitosan-based nanoparticles administration in the experimental hepatotoxicity induced by some antituberculosis drugs—isoniazid and rifampicin—in rats [27].

Erythromycin (ERT) 9-{O-[(2-methoxyethoxy)methyl]oxime} is a macrolide antibiotic clinically used for more than 60 years [28]. ERT acts primarily bacteriostatic against Gram (+) bacteria [29] by inhibiting the production of proteins crucial for bacterial function [30]. It is active in bronchitis, diphtheria, severe campylobacter enteritis, pneumonia, sinusitis, chancroid, syphilis, legionnaires, trench fever, chlamydia, and gonorrhoea [28]. Its gastrointestinal effect may be annoying for patients [31]. Topical applications of ERT have been recommended for the treatment of acne rosacea, acne vulgaris, infections of soft tissue and skin, inflammation of the gums and eyelids [29]. In addition to antimicrobial properties, anti-inflammatory and immuno-modulating effects of ERT have been reported [32,33]. ERT bioavailability is rather low (35%), therefore researches have been made to incorporate ERT stearate in nanosystems, in order to increase its bioavailability and stability to acidic pH [34].

The purpose of our study was the design and structural analysis of original chitosan-coated vesicles entrapping ERT. Furthermore, we aimed to assess these liposomes’ biocompatibility after oral administration in mice, through blood tests and histological examination (liver and kidney).

## 2. Materials and Methods

The protocol of the experimental researches was approved (Certificate No. 24/14.07.2020) and the investigations were carried out in compliance with the recommendations of the Committee for Research and Ethical Issues from ‘Grigore T. Popa’ University of Medicine and Pharmacy from Iasi, Romania, in agreement with the international ethical standards for working with laboratory animals [35].

### 2.1. Materials

L-alpha phosphatidylcholine Egg Yolk specific type XVIE (99% TLC purity), chitosan (from crab shells), chloroform and ERT were purchased from Sigma-Aldrich Chemical Co. (Steinheim, Germany). The chitosan used in the experiment had the following properties: 28% degree of N-de-acetylation, polydispersity index of 3.26, average molecular weight Mw = 309.900 g/mol. Deionized water was obtained using the Model UltraMatic PLUS DI apparatus (Wasserlab, Navarra, Spain).

### 2.2. Laboratory Animals

Healthy, non-genetically modified white mice were used in the experiment (6–8 weeks old, weighing 25–30 g, equally distributed by gender). The animals were purchased from the ‘Cantacuzino’ National Medical-Military Institute for Research and Development, Baneasa Station, Bucharest, Romania, and brought to the bio-base of the University of Medicine and Pharmacy ‘Grigore T. Popa’ Iasi, within the CEMEX (“Centre for Advanced Research and Development in Experimental Medicine”) Laboratory.

The mice were brought one day before the experiment for accommodation, were fed with standard pellets and were kept in specific laboratory conditions: constant temperature of 21 °C ± 2 °C, 50–70% relative humidity, alternating lighting regimen (light/dark ratio = 12 h/12 h). Drinking water was available ad libitum in standardized drinkers for mice; each animal were weighed daily before the start of the experiments and the consumed amount of pellets was measured. During the investigation, the mice were deprived of water and food.

### 2.3. Preparation of Soft Lipid Vesicles

0.1 g Chitosan was dissolved in a 100 mL solution with 1% (*v*/*v*) of acetic acid.

The lipid used was phosphatidylcholine, with a geometric molecular structure that allows it to self-assemble into vesicles in particular conditions [36]. A total of 0.006 g of L-alpha phosphatidylcholine were initially dissolved in 1 mL chloroform; the solvent was removed by evaporation (Rotary evaporator RE-2000A, Ya Rong Biochemical Instrument Factory, Shanghai, China), forming a dry lipid film, which was further dried for 4 h at 50 °C using a vacuum pump. A volume of 12 mL dispersion was prepared by dissolving 176.3 mg ERT in 3.5 mL ethanol (99.8%); water was added up to the total volume. After magnetic stirring for 2 h, this solution was used to hydrate the dry lipid film. Then, ultrasonication was performed for 15 min (field amplitude of 25%/17,668 kJ/30 °C) (Bandelin 2450 SONOPULS ultrasonic homogenizers, Sigma-Aldrich, Steinheim, Germany). Coating the lipid vesicles with chitosan was done by adding 8 mL of 1% chitosan solution in the drug-loaded vesicles dispersion. The vesicle dispersion was subjected to magnetic stirring at 800 rpm for 10 min, and then dialyzed for 2 h for reducing the acidity [37]. Fiber tubular membranes with a pore size of 12,000 Da MWCO type D6191-25EA (Sigma-Aldrich Chemical Co., Steinheim, Germany) were used for dialysis.

### 2.4. Physico-Chemical and Structural Analysis of Lipid Vesicles Loading ERT

The pH values of the solutions were evaluated using a Sartorius Professional PP-50 pH meter (Sartorius Lab Instruments GmbH & Co. KG, Göttingen, Germany). Vesicular ultrasonication was performed with a Sonoplus Bendeline ultrasound generator. The soft lipid vesicles were analyzed in terms of electrophoretic mobility and zeta potential using the Malvern Zetasizer Nano ZS ZEN-3500 (Worchestershire, Worcestershire, UK). To measure electrophoretic mobility, the samples were diluted with 0.1 mM NaCl and then placed in the measuring cell. The zeta potential was calculated with the Smolochowski equation. Each sample was determined in triplicate.

To obtain the calibration curve, ERT was dissolved in ethyl alcohol to obtain the stock solution from which 6 different dilutions were prepared. The UV–vis spectra of the samples were acquired on a Hewlett Packard 8453 UV–VIS spectrophotometer (Waldbronn, Germany).

The spectra were recorded in order to highlight the encapsulation of substances in vesicles and to establish the release profiles.

During the 2 h of dialysis, the organic solvents in the chitosan vesicles loading ERT (ERT-ves) suspension were removed. After centrifugation, the precipitate with ERT-ves erythromycin was dissolved in ethyl alcohol and ERT excluded from the vesicles. The ERT concentration that was loaded into the vesicles could be calculated according to the relation:

Ee (%) = [(Wi − We)/Wi)] × 100,

where Ee is the percentage of efficiency of ERT encapsulation, Wi is the initial mass of ERT, We is the mass of ERT excluded from the vesicles.

Micrographs of chitosan-coated lipid vesicles were obtained by scanning electron microscopy (SEM) (Scanning Electron Microscope SEM EDAX—Quanta 200, Eindhoven, Germany). The measurement of the average diameter of over 2000 vesicles using SEM micrographs was performed using the Image J software Version 1.8.0.

### 2.5. In Vivo Biocompatibility Testing of Lipid Vesicles Loading ERT

Prior to the experiment, the mice were placed on a raised wire mesh under a plexiglass box and allowed to acclimatize to the testing chamber for 2 h. To avoid chronobiological influences, the experiments were performed during the same time interval of the day (8:00 to 0:00). For the in vivo biocompatibility test, 4 groups of 6 animals each were randomly assigned. The mice were treated orally (using an eso-gastric tube), according to the following protocol:

- Group 1 (Control): distilled water 0.1 mL/10 g body weight;
- Group 2 (CHIT): chitosan 0.1 mL/10 g body weight;
- Group 3 (ERT): ERT 50 mg/kg body weight;
- Group 4 (ERT-ves): ERT-ves 50 mg/kg body weight.

The tested drugs were administered in a single dose. Haematological and biochemical investigations were performed at two time points in the experiment: 24 h and 7 days after substances administration, using blood collected from one of the mouse lateral caudal veins [38]. Care was taken not to stress the animals by harvesting, which was very fast (10–15 s) and was performed under local anaesthesia with 1% benzocaine solution [39]. The animal was placed in a Plexiglas tube restraint, and its tail was heated with warm water (at 40 °C), for vein dilation [39,40]. The tail was kept in an extended position, the lateral caudal vein was found at a distance of 3 cm. from the tip and the skin was treated with an antiseptic solution of 70% alcohol (*v*/*v*). The vein was punctured and 0.2–0.3 mL of blood sample were collected; after removing the needle, a gentle pressure was applied locally to stop the eventual bleeding. The collected blood samples were immediately used for laboratory tests.

Biocompatibility testing of lipid vesicles entrapping ERT was based on assessing their effects on the blood count, the serum biochemical tests and on certain immunological parameters.

The Haematology Analyzer 5 DIFF model BF-5180 (DIRUI manufacturer) from PRAXIS Laboratory Iasi was used to assess blood count, activity of liver enzymes such as alanine aminotransferase (ALT), aspartate aminotransferase (AST), the plasma level of lactate dehydrogenase (LDH), serum values of urea and creatinine.

On the 7th day of the experiment, the phagocytic capacity of polymorphonuclear neutrophils in the peripheral blood (PC) was evaluated.

At the end of the experiment, the animals were sacrificed under anaesthesia with 3% isoflurane solution and the peritoneal macrophages were removed by washing the intact peritoneal cavity with 10 mL HANKS solution (37 °C thermostated). The samples were centrifuged (1000 rpm for 10 min), brought into contact with *Staphylococcus aureus* 94 cultures in 0.2% glucose broth, diluted 1:1000 in saline solution, incubated for 48 h at 37 °C and reseeded on culture media. The formation of plate colonies was observed, and serum opsonic capacity (OC) and bactericidal capacity (BC) of peritoneal macrophages were measured.

The liver and kidney fragments were prepared for histopathological examination by fixing the harvested tissues in a 10% formaldehyde solution. The fragments were embedded in paraffin wax, sectioned in thin pieces of 5 μm, and stained with haematoxylin and eosin (H&E). Blades were prepared for microscopic examination and visualized using a Nikon TI Elipse optical microscope (Tokyo, Japan). The images were taken with a Nikon Coolpix 950 digital camera, with a resolution of 1600 × 1200 (1.92 Mpx) and optical zoom × 3.

### 2.6. Statistical Analysis

The obtained data were centralized and statistically processed using the SPSS 17.0 software for Windows 10, using the one-way ANOVA method. The results were presented as the mean values ± standard deviation (S.D.) of mean. The statistical analysis was supplemented with Tukey and Newman–Keuls tests as post hoc tests, for multiple comparisons, that further allow the separation of batches by groups of significance according to the intensity of the effect, therefore achieving the ascending hierarchy of the intensity action of substances. These tests offer the possibility of assessing the significance of the differences recorded in the same group of animals and the variances found between the groups receiving different substances, compared to the control group. The *p* coefficient (probability) value lower than 0.05 was considered statistically significant compared to the control group.

## 3. Results

### 3.1. pH Value of Lipid Solutions with ERT

From Table 1 it can be observed that by adding 1% chitosan, the dispersion of vesicles loaded with ERT has an acidic pH (pH of the ERT-non dialysed-ves solution = 4.00), compared to the pH of the ERT solution (6.12). Moreover, 1% chitosan was used to ensure an increase in positive charge density at the surface of the vesicles leading to improved electrostatic repulsion [41]. Due to the fact that the stabilization of the vesicles with chitosan led to a major decrease in pH, it was necessary to dialysis the solution to obtain a pH value as close as possible to the physiological one, the pH of the ERT-ves sample reaching 6.02.

### 3.2. Size Distribution of Lipid Vesicles with ERT

**Figure 1:**
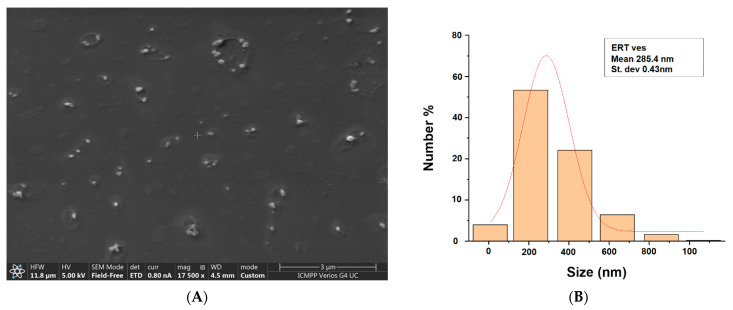
SEM micrograph for ERT-ves (**A**) and the dimensional histogram (**B**) corresponding to the image.

The image in Figure 1A suggests the presence of vesicles with approximately well-dispersed spherical morphology. According to the size distribution obtained by measuring the relative diameters of the vesicles on SEM images, and using the ImageJ software, it was highlighted that the average diameter of ERT-ves is approximately 284.5 nm (Figure 1B).

**Figure 2:**
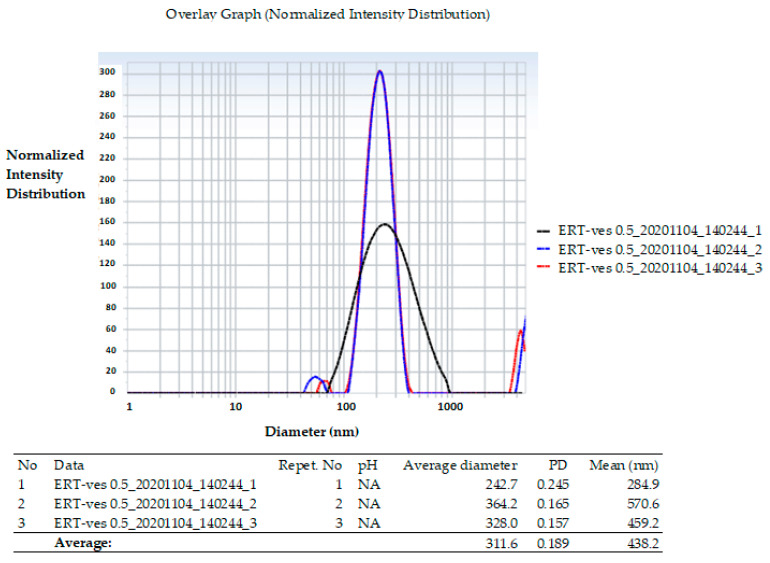
Diameter distribution of lipid vesicles loading ERT.

On the other hand, the average hydrodynamic size of the vesicles obtained by the Malvern Zetasizer Nano ZS ZEN-3500 analysis, showed that the ERT-ves has an average value of 311.6 nm (Figure 2).

The polydispersity index Pi of 0.189 nm suggests the presence of relatively homogeneous vesicles, according to the dimensional histogram.

### 3.3. Zeta Potential of Lipid Vesicles with ERT

**Figure 3:**
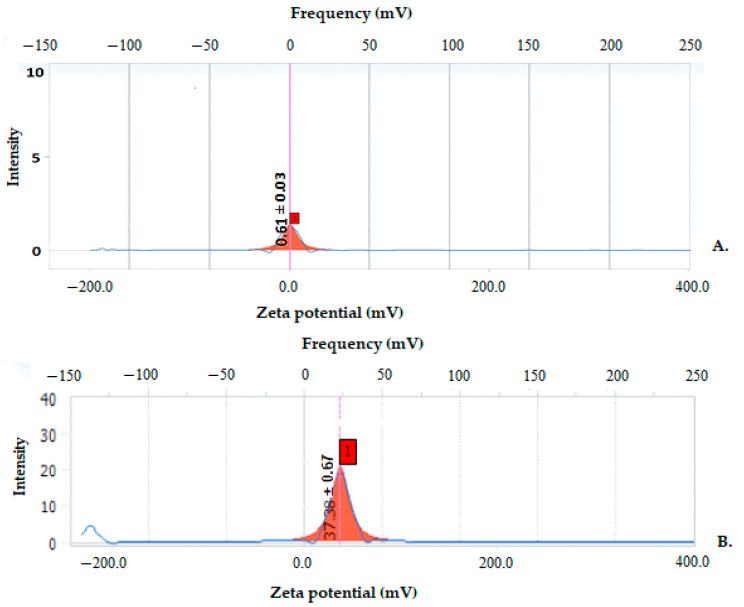
Zeta potential of chitosan-free ERT vesicles (**A**) and chitosan-coated ERT vesicles (**B**).

To predict the long-term physical stability of colloidal systems, the Zeta potential was evaluated. The stability conditions were analyzed in relation to a Zeta potential measurement for positive spherical particles dispersed in a homogeneous medium. In general, Zeta potential values higher than 30 mV indicate a long-term stability of the aqueous dispersion. From the distribution of the Zeta potential, we can conclude that all the obtained vesicle systems were mainly positively charged. Vesicles with ERT without chitosan showed a Zeta potential of 0.16 mV indicating a low degree of stability. Coating with chitosan the surface of ERT-loaded vesicles resulted in a Zeta potential of 37.38 mV, indicating an increase in suspension stability (Figure 3).

### 3.4. Efficiency of Drug Uptake

**Figure 4:**
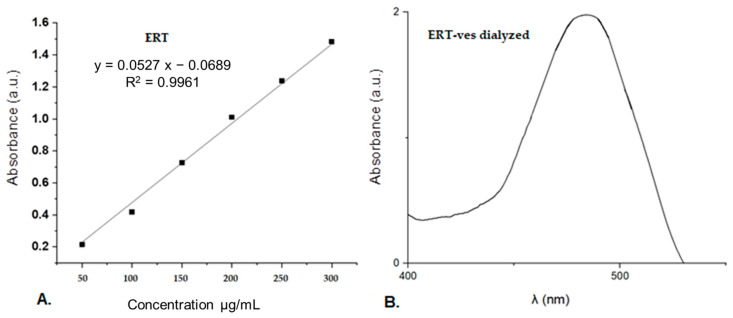
Calibration curve (**A**) of ERT solution and the absorption spectra (**B**) of ERT-ves.

To determine the drug concentration released from the samples, a calibration curve for ERT in ethyl alcohol was performed for which a maximum absorption at 482 nm was obtained. The ERT concentration used to obtain the calibration curve was 292.9 mg/mL (Figure 4).

To estimate the percentage of drug incorporated into chitosan-coated vesicles, the ERT-ves suspension, with a volume of 23.5 mL, previously dialyzed, was treated with chloroform 1: 100, then centrifuged at 5000 rotations per minute, during 45 min. The absorption spectrum of the vesicular ERT content was compared with the spectrum of the free ERT solution, for the same absorption peak. The experiment was performed in triplicate. Given that the initial mass of ERT was 7.66 mg/mL, and that of ERT excluded from vesicles 3.437 mg/mL, the calculated percentage efficiency of drug incorporation into vesicles was 55.13%.

### 3.5. In Vivo Biocompatibility Evaluation Hematological Tests

The results obtained after quantification of erythrocytes (GR), hemoglobin (Hb), hematocrit (Ht), and leukocyte formula [polymorphonuclear neutrophils (PMN), lymphocytes (Ly), eosinophils (E), monocytes (M), basophils (B)], in animals that were treated with the nanovesicles entrapping ERT are presented in Table 2 and Table 3.

There were no statistically significant changes in erythrocyte counts, haemoglobin (Hb), haematocrit (Ht), in animals receiving the solution of vesicles with CHIT, ERT, or chitosan-based lipid vesicles loaded with ERT compared to animals from the control group, at 24 h and 7 days in the experiment (Table 2).

There were revealed no statistically significant variations in serum Hb values in animals treated with CHIT, ERT, or ERT-ves dispersion compared to animals that received distilled water after one day and after 7 days in the experiment (Table 2).

The percentage values of the PMN, Ly, E, M, and B determined from animals receiving CHIT and ERT were comparable to those from animals receiving distilled water (Table 3). No significant variations between the percentage values of the leukocyte formula (PMN, Ly, E, M, B) from mice treated with ERT-ves and those from control animals were noted (Table 3).

### 3.6. Liver Enzyme Activity

Haematological investigations did not reveal significant variations in AST values in the groups to which CHIT, ERT, ERT-ves were administered compared to the control group at any time point of the determinations (Table 4). The using of CHIT, ERT, and ERT-ves did not produce substantial changes in ALT activity compared to the distilled water group, after 24 h and 7 days, respectively. A significant increase (** *p* < 0.01) of LDH values was observed in the group of mice treated with chitosan vesicles (1904.33 ± 264.80) compared to the group treated with distilled water (1250.88 ± 167.04) after 7 days in the experiment (Table 4). The ERT administration produced a considerable increase (* *p* < 0.05) in LDH values (1619.333 ± 198.032) compared to the group that received distilled water (1250.883 ± 167.044) after 7 days (Table 4). This observed increase of LDH levels, although statistically significant, was not associated with the intensification of AST, ALT activity or with structural alterations of liver tissue, for the same animals groups.

### 3.7. Serum Levels of Urea and Creatinine

No substantial changes in serum urea plasma levels were detected in animals treated with CHIT, ERT, ERT-ves, compared to animals treated with distilled water, after 24 h and 7 days in the experiment (Table 5). The use of CHIT, ERT, was not associated with obvious variations in blood creatinine values compared to the control group at both time points of the determination (Table 5). ERT-ves treatment was followed by a significant decrease in creatinine values * *p* < 0.05, compared to the control, at the same time (7 days) in the experiment (Table 5).

### 3.8. Activity of Immunological Biomarkers

No significant variations in OC in mice that received CHIT, ERT, ERT-ves were found when compared to animals in the group with distilled water, both after 24 h and after 7 days of administration (Table 6). No notable changes were observed regarding PC in the animal groups treated with CHIT, ERT, ERT-ves compared to the control group at both time points of the investigation (Table 6). No significant differences in BC values were observed in animals that were administered CHIT, ERT, and ERT-ves compared to the group treated with distilled water after 24 h or after 7 days in the experiment (Table 6).

### 3.9. Hepatic Histopathological Examination

**Figure 5:**
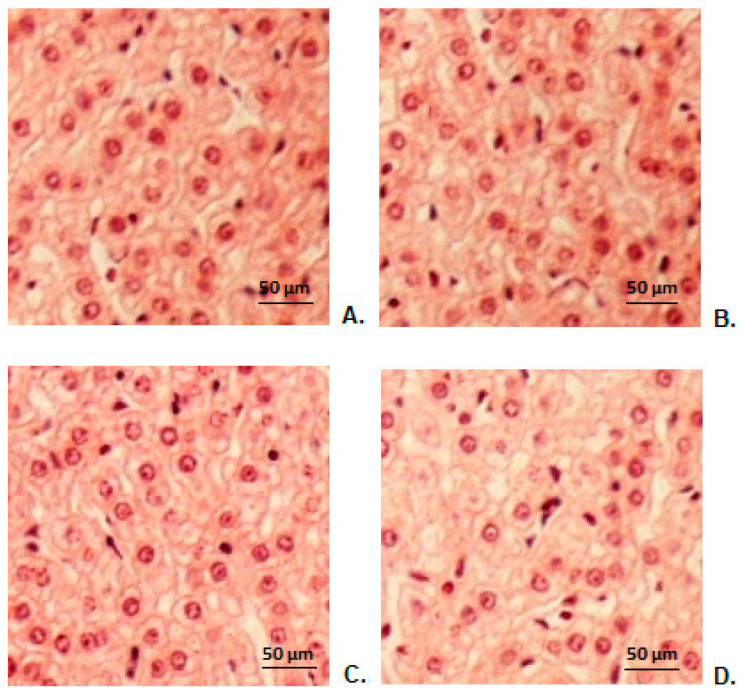
Histopathological images of hepatic architecture in animals treated with distilled water (**A**), CHIT (**B**), ERT (**C**), ERT-ves (**D**). (H&E stain × 20).

The histopathological study of liver fragments harvested from animals in the control group showed a normal hepatic conformation (Figure 5A). The histopathological examination did not show substantial changes in the hepatic architecture in animals treated with CHIT (Figure 5B), ERT (Figure 5C), ERT-ves (Figure 5D) compared to the group that received distilled water (Figure 5A).

### 3.10. Renal Histopathological Examination

**Figure 6:**
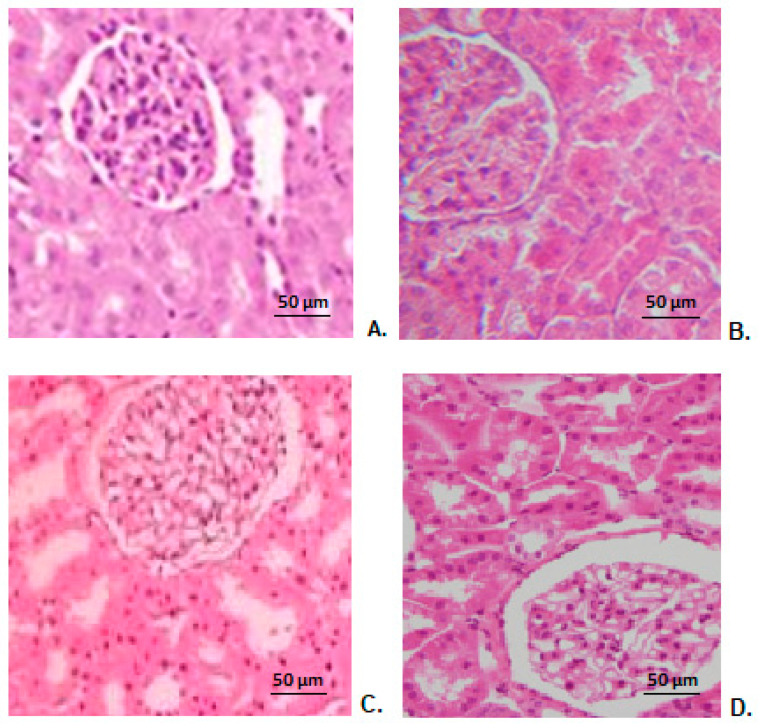
Histopathological images of kidney architecture in animals treated with distilled water (**A**), CHIT (**B**), ERT (**C**), ERT-ves (**D**) (H&E stain × 20).

Microscopic histopathological examination of kidney fragments taken from mice treated with distilled water showed a normal renal architecture (Figure 6A). No obvious alterations of renal structure were found in animals receiving CHIT (Figure 6B), ERT (Figure 6C), ERT-ves (Figure 6D) compared to those from the control group (Figure 6A).

## 4. Discussions

Approaches to nanoantibiotics have been made for increasing ERT efficacy, by entrapping it in solid lipid nanoparticles, which showed increased efficacy, good accumulation into bacteria cells but limited stability (48 h to a maximum of three months) [28,42]. In another study, in vitro characterization and antimicrobial evaluation were performed for ERT-loaded solid lipid nanoparticles gel compared to ERT plain gel. It was registered a sustained delivery of drug from the novel formulation enhancing the anti-infective activity after 30 h [43]. ERT and gentamicin were encapsulated into liposomes composed of 1,2-Dipalmitoyl-sn-glycero-3-phosphocholine (0.11382 g/mL) and cholesterol (0.01 g/mL) at a molar ratio of 6:1 with increased activity on the *Pseudomonas aeruginosa*, diminishing the minimum inhibitory and bactericidal concentrations by 4–32 fold overall [44].

In our study, we encapsulated hydrophilic erythromycin (as an aqueous solution) in lipid vesicles that were stabilized with 1% chitosan. Comparing the resulted histogram (micrographic and hydrodynamic) we found the vesicles dimensions about 311 nm, and the average hydrodynamic ERT-ves size around 284 nm assessed by SEM analysis. The same dimensional range has been reported by other authors for small (200–350 nm) multilamellar liposomes of phosphatidylglycerol, with various fatty acids composition, entrapping ERT and azithromycin, but no data about liposomes based on phosphatidylcholine are available [45]. Furthermore, the gel-based lipid systems measuring 176.2 and 374 nm were obtained for the release of ERT with antimicrobial efficacy on *Pseudomonas aeruginosa*. [43]. Other researchers have used a dehydration-rehydration method to produce azithromycin-loaded liposomes based on dipalmitoyl-sn-glycero-3-phosphocholine and cholesterol, with an average diameter of 406.07 ± 45 nm [46].

Determination of Zeta potential represents a significant technique for characterizing nanovesicles in order to estimate surface load, which can further be used to understand the physical stability of nanodispersions [47]. The 0.16 mV value of the Zeta potential of the ERT-ves without chitosan leads to the aggregation and flocculation of the particles due to the van der Waals attraction forces acting on the particles, thus to physical instability [48]. The positive value of 37.38 mV of the Zeta potential for ERT-ves indicates a good physical stability of the nanodispersions due to the electrostatic repulsion of the individual particles [49].

Several studies have found that chitosan coating improves the stability of soft lipid vesicles by forming a wall that prevents swelling and release of the encapsulated drug (e.g., natural compounds, anti-inflammatory drugs, antimicrobials, hormonal medication, cardiovascular medication) [50,51].

Chitosan is a positive linear polysaccharide that can form stable complexes with negative compounds, such as phospholipids, and can be a candidate for drugs encapsulation and their controlled release [52]. The electrostatic attraction forces of chitosan [53] act on the negative surface charge of the lipid bilayer loaded with ERT. The low Zeta potential of drug-loaded but non-dialyzed lipid vesicles suggests that the solution has low stability near the dispersion threshold. It is known that the lipid bilayer is negatively charged. During the process of coating the lipid vesicles with chitosan, the electrostatic interactions between the positive charges of the polymer and the negative surface of the lipid bilayer occur. It is reported that electrostatic interactions are stronger than the binding energy of hydrogen bonds, which are formed between polysaccharides and phospholipid head groups [51]. Moreover, it seems that the van der Waals forces, which are weaker than hydrogen bonds, also contribute to self-assembly processes [54]. Using phosphatidylcholine, which is a neutral lipid to obtain lipid vesicles, the coating with chitosan only increases the positive charge on the surface of the vesicle. The increase of the Zeta potential leads to the intensification of the electrostatic repulsion forces between ERT-ves while maintaining the stability of the suspension.

Maintaining the suspensions transparent for more than six months after preparation, at room temperature, may be due to the optimal coating with chitosan 1% which can protect the phospholipid membranes from oxidation during storage at different temperatures [55]. The calibration curve indicates that the absorption of ERT is linear. ERT completely extracted from vesicles by centrifugation showed the same absorption peak at 482 nm as unencapsulated ERT. Much of the aqueous ERT was entrapped inside the lipid vesicles with 55.13% efficiency.

After the preparation of ERT-ves, we evaluated their biocompatibility using the assessment of some serum parameters. The in vivo studies are preliminary investigations, focused on highlighting the impact of the administration of such original systems on some plasma constants, which are relevant elements for estimating the existence of an inflammatory reaction, of liver and kidney function disturbances, of oxidative processes, and organs structural alterations.

In our experimental conditions, the laboratory analysis did not show marked changes in the number of red blood cells and at the hematocrit values, nor of the percentage of leukocyte formula elements, between the animals treated with CHIT, ERT, nor with ERT-ves, versus control group, at any time point of the determinations. The measuring of the liver enzymes activity, the basic modality to appreciate the functional state of the liver, did not demonstrate important dissimilarities in ALT and AST values between the CHIT, ERT, ERT-ves groups and distilled water group during the experiment, thus suggests that their use did not influence the liver functional capacity. The use of CHIT and ERT was accompanied by an obvious increase in LDH activity compared to control, seven days after their administration, this modification being not correlated with increased serum transaminases, nor with histopathological detectable structural alterations of liver tissue.

The measurement of urea and creatinine levels was also performed, these being useful elements for assessing the functional capacity of the kidneys. It was observed that, the oral administration of the test substances was not accompanied by obvious variations in the serum levels of these two parameters, compared to the group treated with distilled water, throughout the experiment, these findings being relevant elements for the lack of their renal toxicity.

The phagocytic and bactericidal functions of polymorphonuclear leukocytes and the opsonic activity of serum from mice treated with CHIT, ERT and ERT-ves, were not significantly influenced during the experiment, which makes us appreciate that these substances do not interfere with the animals immune defense capacity.

Observing that the administration of CHIT, ERT and ERT-ves was not associated with substantial variations of the oxidative stress markers, compared to the distilled water group, we can consider that these substances do not produce notable oxidative damage in the period of evaluation.

Our laboratory investigations were supplemented by hepatic and renal histopathological examination, which did not detect obvious changes in liver structure or kidney conformation in animals in the CHIT, ERT, ERT-ves groups compared to the control group, findings that are consistent with the biochemical results used to assess the functional integrity of these organs.

## 5. Conclusions

In the current context of the alarming increase in antibiotic resistance, we have obtained original formulations for ERT liposomes. Their oral administration did not produce sizeable modifications in the percentages of the leukocyte formula elements, of some blood constants useful for evaluating the hepatic and renal function, respectively of some markers of oxidative stress and immune system activity, which suggests a good biocompatibility in mice. The laboratory investigations were supplemented by histological examination, which did not reveal significant alterations of liver and kidney architecture in mice treated with chitosan nanovesicles entrapping ERT, compared with control group animals. We can appreciate the research should be continued and that these nanosystems could have medical applicability.

## Figures and Tables

**Table 1:** pH values of suspensions containing ERT.

Solution	pH
Erythromicyn vesicles (ERT)	6.12
Erythromycin vesicles in chitosan (before dialysis) (ERT-non dialysed-ves)	4.00
Erythromycin vesicles in chitosan (after dialysis) (ERT-ves)	6.02

**Table 2:** Blood erythrocyte, Hb and Ht levels in animals treated with CHIT, ERT, ERT-ves (data are expressed as arithmetic mean ± S.D. of mean values for 6 animals per group).

	Point in Time	GR (mil/μL)	Hb (g/mL)	Ht (%)
Control	24 h	8.50 ± 1.05	12.67 ± 2.58	43.67 ± 2.73
	7 days	9.77 ± 0.14	15.12 ± 0.57	41.48 ± 1.32
CHIT	24 h	9.34 ± 0.57	15.41 ± 0.25	41.50 ± 0.14
	7 days	9.79 ± 0.10	15.67 ± 0.38	42.01 ± 0.57
ERT	24 h	9.80 ± 0.73	15.37 ± 1.58	41.52 ± 0.70
	7 days	9.82 ± 0.25	15.40 ± 0.42	41.55 ± 2.38
ERT-ves	24 h	9.59 ± 1.28	15.24 ± 0.73	40.69 ± 3.14
	7 days	9.64 ± 0.16	15.23 ± 0.42	40.72 ± 0.67

**Table 3:** Leukocyte formula in animals treated with CHIT, ERT, ERT-ves (data are expressed as the arithmetic mean ± S.D. of mean values for 6 mice per group).

	Point in Time	Leucokyte Formula				
		%				
		PMN	Ly	E	M	B
Control	24 h	19.72 ± 2.42	77.83 ± 4.59	0.47 ± 0.26	1.78 ± 1.23	0.20 ± 0.07
	7 days	19.73 ± 2.95	77.86 ± 3.72	0.52 ± 0.48	2.05 ± 1.81	0.22 ± 0.13
CHIT	24 h	18.74 ± 2.87	79.26 ± 4.42	0.57 ± 0.52	1.23 ± 1.17	0.20 ± 0.03
	7 days	18.73 ± 1.91	80.88 ± 1.82	0.72 ± 0.85	1.45 ± 1.22	0.20 ± 0.17
ERT	24 h	19.44 ± 3.23	77.33 ± 4.86	0.53 ± 0.42	2.45 ± 1.07	0.25 ± 0.07
	7 days	19.35 ± 3.74	76.05 ± 5.42	0.57 ± 0.71	2.98 ± 1.25	0.28 ± 0.20
ERT-ves	24 h	18.75 ± 3.23	78.97 ± 4.59	0.56 ± 0.59	1.47 ± 0.26	0.25 ± 0.13
	7 days	18.77 ± 2.42	79.45 ± 2.84	0.55 ± 0.52	0.95 ± 0.52	0.27 ± 0.23

**Table 4:** ALT, AST, and LDH levels in animals treated with CHIT, ERT, ERT-ves (data are expressed as arithmetic mean ± S.D. of mean values for 6 animals per group).

	Point in Time	ALT (U/mL)	AST (U/mL)	LDH (U/L)
Control	24 h	40.30 ± 3.19	120.50 ± 19.29	1254.17 ± 165.33
	7 days	43.33 ± 3.62	119.00 ± 18.17	1250.88 ± 167.04
CHIT	24 h	42.17 ± 5.21	159.33 ± 35.20	1311.27 ± 249.19
	7 days	46.83 ± 6.43	161.17 ± 37.62	1904.33 ± 264.80 **
ERT	24 h	39.30 ± 6.17	120.50 ± 18.43	1302.13 ± 101.33
	7 days	37.8 ± 4.38	117.17 ± 12.29	1689.00 ± 96.49 **
ERT-ves	24 h	39.67 ± 5.04	140.50 ± 28.33	1398.17 ± 185.43
	7 days	41.00 ± 5.33	142.00 ± 31.71	1407.67 ± 294.90

** *p* < 0.01 compared to control group.

**Table 5:** Urea and creatinine levels in animals treated with CHIT, ERT, ERT-ves (data are expressed as arithmetic mean ± S.D. of mean values for 6 animals per group).

	Point of Time	Urea (mg/mL)	Creatinine (mg/dL)
Control	24 h	47.83 ± 6.43	0.38 ± 0.02
	7 days	45.00 ± 5.87	0.39 ± 0.02
CHIT	24 h	46.67 ± 1.37	0.38 ± 0.02
	7 days	42.33 ± 4.03	0.37 ± 0.03
ERT	24 h	45.83 ± 1.94	0.38 ± 0.01
	7 days	42.67 ± 8.31	0.37 ± 0.01
ERT-ves	24 h	49.33 ± 3.14	0.37 ± 0.01
	7 days	50.67 ± 2.07	0.37 ± 0.03

**Table 6:** Effects of CHIT, ERT, ERT-ves on OC, PC, and BC values in mice (values are presented as the arithmetic mean ± S.D. of mean values for 6 animals per group).

	Point of Time	OC (Bacterial Colonies/mL)	PC (Bacterial Colonies/mL)	BC (Bacterial Colonies/mL)
Control	24 h	808.33 ± 25.00	531.50 ± 15.60	715.67 ± 10.76
	7 days	787.17 ± 30.80	529.67 ± 13.02	712.33 ± 9.69
CHIT	24 h	805.40 ± 29.73	527.43 ± 14.48	713.55 ± 9.80
	7 days	794.43 ± 29.58	530.67 ± 14.55	719.40 ± 11.48
ERT	24 h	811.21 ± 29.47	530.83 ± 15.05	715.33 ± 11.58
	7 days	791.17 ± 29.53	532.55 ± 14.03	717.67 ± 13.47
ERT-ves	24 h	806.67 ± 29.76	530.60 ± 14.58	717.55 ± 16.33
	7 days	799.17 ± 30.11	533.21 ± 13.24	719.17 ± 17.48

## Data Availability

The data is contained in the manuscript.
